# Butyrate reduces epithelial barrier dysfunction induced by the foodborne mycotoxin deoxynivalenol in cell monolayers derived from pig jejunum organoids

**DOI:** 10.1080/19490976.2024.2430424

**Published:** 2024-11-21

**Authors:** Julie Alberge, Eloïse Mussard, Carine Al-Ayoubi, Corinne Lencina, Christelle Marrauld, Laurent Cauquil, Caroline S. Achard, Ivan Mateos, Imourana Alassane-Kpembi, Isabelle P. Oswald, Laura Soler, Sylvie Combes, Martin Beaumont

**Affiliations:** a GenPhySE, Université de Toulouse, INRAE, ENVT, Castanet-Tolosan, France; b Lallemand Animal Nutrition, Blagnac Cedex, France; c Toxalim (Research Center in Food Toxicology), Université de Toulouse, INRAE, ENVT, INP-Purpan, Toulouse, France; d Departamento de Producción Animal, Universidad de León, León, Spain; e Centre de recherche en infectiologie porcine et avicole (CRIPA), Faculté de médecine vétérinaire, Université de Montréal, Saint-Hyacinthe, QC, Canada

**Keywords:** Gut microbiota, metabolites, toxin, epithelium, permeability, enteroids

## Abstract

The foodborne mycotoxin deoxynivalenol (DON) produced by *Fusarium* species threats animal and human health through disruption of the intestinal barrier. Targeting the gut microbiota and its products appears as a promising strategy to mitigate DON intestinal toxicity. In this study, we investigated whether the bacterial metabolite butyrate could alleviate epithelial barrier disruption induced by DON. We used a model of cell monolayers derived from porcine jejunum organoids allowing to reproduce the cellular complexity of the intestinal epithelium. Our results show that DON dose-dependently disrupted the epithelial barrier integrity, reduced epithelial differentiation, and altered innate immune defenses. Butyrate attenuated the DON-induced increase in paracellular permeability. Butyrate also prevented epithelial barrier dysfunction triggered by anisomycin, a ribosome inhibitor like DON. Moreover, butyrate partially counteracted the effects of DON on tight junctions (TJP1, OCLN), innate epithelial defenses (PTGS2, CD14, TLR4, TLR5), and absorptive cell functions (CA2, VIL1, NHE3, CFTR). In contrast, butyrate did not prevent the toxic effects of DON on mitochondrial metabolism, proliferation and goblet cell functions. Taken together, our results demonstrate that the bacterial metabolite butyrate is able to reduce DON-induced epithelial barrier disruption.

## Introduction

Deoxynivalenol (DON) is a trichothecene mycotoxin produced by fungal species of the *Fusarium* genus.^1^ DON frequently contaminates cereals (wheat, barley and corn) and is a major cause of food poisoning, which threatens both human and animal health.^2^ The small intestinal epithelium is highly exposed to this foodborne mycotoxin which inhibits protein synthesis through its binding to ribosomal peptidyl transferases.^3^ DON-induced ribotoxic stress contributes to disrupt epithelial barrier dysfunction by altering tight junctions.^4–6^ DON also reduces epithelial differentiation,^7–9^ impairs nutrient absorption,^10^ depletes goblet cells,^11,12^ reduces the production of antimicrobial peptides,^13^ alters the production of inflammatory mediators^14–16^ and inhibits proliferation.^13,17^ Despite improvements in agricultural practices, contamination of cereals with DON cannot be avoided yet and could rise in the future due to global warming.^18^ Thus, the development of strategies reducing the intestinal toxicity of DON is required.

The gut microbiota is emerging as a novel player able to mitigate the toxicity of mycotoxins.^19,20^ For instance, intestinal bacteria can metabolize DON into de-epoxy DON (DOM-1), which is less toxic for the gut barrier.^21,22^ The exposure of epithelial cells to DON can also be reduced through its binding to the cell walls of commensal bacteria and yeasts.^20^ Moreover, the gut microbiota may reduce the toxicity of mycotoxins by enhancing the gut barrier function, notably through the production of bacterial metabolites.^23^ Butyrate (BUT) is a metabolite produced by the gut microbiota mainly through fiber fermentation and is considered protective for the intestinal epithelium.^24,25^ BUT improves the epithelial barrier function,^26–29^ promotes differentiation of secretory and absorptive epithelial cells^29,30^ and increases the expression of antimicrobial peptides.^31,32^ Thus, BUT appears to be a promising metabolite to prevent the detrimental effects of DON on the intestinal epithelium. Indeed, BUT restored epithelial barrier function and mucus secretion in the human goblet-like cell line HT29-MTX exposed to DON.^33^ BUT also alleviated DON-induced barrier dysfunction and restored antimicrobial peptide production in the porcine enterocyte cell line IPEC-J2.^34,35^ However, these results were obtained in cell lines that present genomic abnormalities and do not reproduce the cellular diversity of the intestinal epithelium. This is a major limitation, as the effects of both BUT and DON were shown to be cell type specific and modulated by the level of epithelial differentiation.^9,24^

Here, we aimed to investigate the potential protective effects of BUT against DON toxicity in a pig jejunum organoid model reflecting the cellular complexity of the intestinal epithelium.^36^ In addition, organoid epithelial cells lack genomic abnormalities because they are derived directly from crypt stem cells. The pig was chosen as this species is highly sensitive to DON^11^ and is an appropriate model to study the human intestine.^37^ The jejunum was selected since this intestinal region is highly exposed to DON and as pig organoids were shown to retain a gut location-specific phenotype.^38,39^ Pig jejunum organoid cells were cultured as monolayers in order to expose epithelial cells to BUT and DON at their apical side, as observed *in vivo*.^40,41^ Moreover, this culture format allowed us to evaluate the transport of BUT and DON through the epithelium and their effects on the barrier function.

## Materials and methods

### Culture of pig jejunum organoids

Jejunum organoids derived from suckling piglets (21-day-old) were obtained from our in-house biobank and cultured as described before.^39,41^ Briefly, frozen organoids kept in liquid nitrogen were thawed at 37°C, centrifuged (500 g, 4°C, 5 min) and seeded in Matrigel (Corning, cat#354234) in a pre-warmed 48-well plate (25 µL/well). Organoid culture medium containing IntestiCult Organoid Growth Medium (Human) (StemCell Technologies, cat#6010) supplemented with 1% Penicillin-Streptomycin (Sigma, cat#P4333) and 100 µg/mL Primocin (InvivoGen, cat#ant-pm-05) was added (250 µL/well). Organoids were cultured at 37°C with 5% CO_2_. Two or 3 days after seeding, organoids in Matrigel were washed in PBS (ThermoFischerScientific, cat#10010015) and homogenized by pipetting in warm TrypLE (ThermoFischerScientific, cat#12605-010) before incubation for 15 min at 37°C. Digestion was stopped by adding cold complete DMEM (DMEMc) containing DMEM (ThermoFischerScientific, cat#31966047) supplemented with 10% fetal bovine serum (FBS, ThermoFischerScientific, cat#10270-106) and 1% Penicillin-Streptomycin. Cells were centrifuged (500 g, 4°C, 5 min) and counted using a Countess 3 Automated Cell Counter (ThermoFischerScientific, cat#16842556). Organoid cells were seeded in Matrigel:DMEMc (v/v: 2:1) in pre-warmed 24-well plates (3 000 cells/50 µL/well) and organoid culture medium was added (500 µL/well) and replaced every 2–3 days. Organoids were used to seed cell monolayers 5 days after seeding. Organoids were used between passages 3 and 9. For cryopreservation, organoids in Matrigel were harvested by pipetting in a freezing solution containing 80% DMEMc, 10% FBS, 10% DMSO (Corning, cat#25-950-CQC) and transferred in a cryotube placed at −80°C in a CoolCell™ LX Cell Freezing Container (Corning, cat# 432003) before long-term storage in liquid nitrogen.

### Culture of cell monolayers derived from pig jejunum organoids

Cell culture inserts for 24-well plates (Corning, cat#353095) were coated with 50 µg/mL Collagen type IV from human placenta (Sigma, cat#C5533) for 2 h at 37°C (150 µL/well). The coating solution was removed and the inserts were dried for 10 min by opening the plate lid under the cell culture cabinet. Organoids (5-days after seeding) were dissociated and cells were counted and centrifuged as described above. Cells were resuspended in organoid culture medium supplemented with 20% FBS and 10 µM Y27632 (ATCC, cat#ACS-3030) before seeding in inserts (2.5 10^5^ cells/insert). The same medium was used at the basal side. Cell culture inserts were placed in a 24-well plate (apical volume: 200 µL, basal volume: 500 µL) or in a CellZscope+ system (nanoAnalytics) used for automatic measurement of transepithelial electrical resistance (TEER, apical volume: 400 µL, basal volume: 770 µL). Cells were incubated at 37°C, 5% CO_2_.

### Treatments of cell monolayers derived from pig jejunum organoids

One day after seeding, cell monolayers derived from pig jejunum organoids were washed with PBS and the apical medium was replaced by PBS (control) or 1 mM sodium butyrate (BUT, Sigma, cat#B5887) or 1 µM trichostatin A histone deacetylase inhibitor (TSA, Sigma, cat#T1952). The epithelial barrier integrity was not affected by the replacement of the apical medium by PBS, as indicated by the measurement of stable and high TEER values (>1000 Ohm.cm^2^). Two days after seeding, cell monolayers were washed with PBS and the apical medium was replaced with PBS (control) or 1 mM BUT or 1 µM TSA or 5–100 µM DON (Sigma, cat#D0156) or 1 µM anisomycin (ANI, Sigma, cat#A5862) or 1 mM BUT and 100 µM DON (BUT+DON) or 1 mM BUT and 1 µM ANI (BUT+ANI) or 1 µM TSA and 100 µM DON (TSA+DON). The basal medium was replaced daily with organoid culture medium supplemented with 20% FBS. Three days after seeding, the apical and basolateral media were collected and stored at −20°C. Paracellular permeability was evaluated as described below. For gene expression analysis, cells were lyzed in 300 µL TriReagent (Ozyme, cat# ZR2050-1-200) and kept at −80°C until RNA purification. For protein analysis (Western Blot), cells were lyzed in RIPA buffer (Thermo Fisher Scientific, cat#89901) supplemented with cOmplete™ protease inhibitor cocktail (Sigma, cat#11697498001) and kept at −80°C until analyses. Each experiment was repeated with jejunum organoid cell monolayers derived from at least three 21-day-old suckling piglets.

### Permeability assay

FITC-dextran 4 kDa (Sigma, cat#FD4-100 MG) prepared in warm HBSS (Thermo Fisher Scientific, cat#15266355) was added at the apical side (2.2 mg/mL, 200 µL). Warm HBSS was added at the basal side (500 µL). After incubation (2 h, 37°C), fluorescence (Excitation: 495 nm, Emission: 530 nm) of the basal medium was quantified with a multimode plate reader Infinite 200 PRO (Tecan).

### Lactate dehydrogenase assay

Cytotoxicity was evaluated by quantification of lactate dehydrogenase (LDH) release in the apical culture medium by using the LDH CyQUANT™ kit (Thermo Fisher Scientific, cat#C20300), following the manufacturer instructions.

### Gene expression analysis

RNA was purified by using the Direct-zol RNA Microprep kit (Zymo Research, cat#R2062), following the manufacturer instruction. RNA was eluted in 15 µL RNAse-free water and quantified with a NanoDrop 8000 spectrophotometer (Thermo Fisher Scientific). RNA (300 ng) were reverse transcribed to cDNA by using GoScript Reverse Transcription Mix, Random primer (Promega, cat#A2801), following the manufacturer instructions. Gene expression was analyzed by real-time qPCR using QuantStudio 6 Flex Real-Time PCR System (Thermofisher) or Biomark microfluidic system using 96.96 Dynamic Arrays IFC for gene expression (Fluidigm) according to the manufacturers recommendations. The sequences of the primers used are presented in supplementary table S1. Data were normalized to the stably expressed gene RPL32 and analyzed with the 2^−ΔCt^ method.

### Western blot

Protein concentration was measured in cell lysate by using the Pierce™ BCA Protein Assay kit (Thermo Fisher Scientific, cat#23225), according to the manufacturer instruction. Immunoblotting was conducted as previously described.^42^ In brief, total proteins (15 µg) were separated on 4.5–12.5% acrylamide-bisacrylamide gradient gels (Bio-Rad) and electrotransferred onto nitrocellulose membranes (Millipore). The membranes were then blocked with RotiBlock (Carl Roth GmbH) for one hour to avoid nonspecific binding sites. Next, membranes were incubated with rabbit primary antibodies specific for Claudin-3 (CLDN3, Thermo Fisher Scientific, #34-1700) or Occludin (OCLN, Thermo Fisher Scientific, #71-1500) diluted 1:250, under agitation, overnight at 4°C. Following washing, membranes were incubated with fluorescent goat anti-rabbit IgG secondary antibodies (dilution 1:10 000) (Biotium) for 1 h, and fluorescence detection was performed using an Odyssey Imaging System (Li-Cor Biosciences). After image digitization, band intensities were quantified using Image Studio Lite Software v5.2 (Li-Cor Biosciences). For normalization, values in each lane were adjusted relatively to the corresponding total protein stain, quantified with Revert™ 700 Total Protein Stain (Li-Cor Biosciences). This approach is recognized as being equally sensitive and, in many cases, more specific compared to the use of highly abundant proteins like β-actin or GAPDH.^43^

### Metabolomics

Culture media (apical and basal) were centrifuged (18 000 g, 10 min, 4°C). The supernatant was collected and 50 µL was mixed with 600 µL of phosphate buffer pH 7 prepared in D_2_O and containing the internal standard TSP (1 mm). Samples were transferred in 5-mm nuclear magnetic resonance (NMR) tubes. The spectra were acquired at 300 K using the Carr-Purcell-Meiboom-Gill (CPMG) spin-echo pulse sequence with pre-saturation on a AVANCE III HD NMR spectrometer operating at 600.13 MHz for 1 h resonance frequency using a 5 mm inverse detection CryoProbe (Bruker) at the metabolomics platform MetaToul-AXIOM (INRAE, Toulouse, France) as described before.^44^ The area under the curve of signals at 0.89 ppm (triplet) and 1.09 ppm (singulet) were calculated with the TopSpin software (v4.1.4, Brucker) to quantify BUT and DON, respectively. These signals were selected by comparison with spectra of pure compounds.

### Confocal imaging

Click-iT EdU Imaging Kit (Thermo Fisher Scientific, cat# C10339) was used to quantify proliferative cells, following the manufacturer instructions. Cells were incubated with EdU (10 µM) for 2 h at 37°C. Fixation, permeabilization and staining was performed as described previously.^41^ Actin was stained with Phalloidin-TRITC (Sigma, cat# P1951). OCLN was detected with a polyclonal rabbit antibody (Thermo Fisher Scientific, cat#71-1500, working dilution 1:200) and a goat anti-rabbit IgG antibody (Thermo Fisher Scientific, cat#A-11008, working dilution 1:1000). DNA was stained with DAPI (Thermo Fisher Scientific, cat#D1306, 5 µg/mL). Fluorescence staining was analyzed with a confocal laser scanning microscope TCS SP8 (Leica). Images were acquired in the sequential mode using LAS X software (Leica).

### Animal experiment

The experiment was performed in the animal facility of INRAE TOXALIM (Toulouse, France). All procedures were performed in accordance with the European Directive on the protection of animals used for scientific purposes (Directive 2010/63/EU) and validated by the local ethics committee for animal experiments Toxcomethique (APAFIS#8280-2016122010097752v3). Twenty crossbred castrated male piglets weaned at 28 days of age were acclimatized for one week in the animal facility before receiving for 28 days either a basal diet (*n* = 10) or a DON-contaminated diet (2.82 mg DON/kg of feed, *n* = 10). The diet composition, piglet growth, plasma biochemistry, plasma metabolome and histological parameters were described before.^45^ Fecal samples were collected from the rectum weekly and kept at −80°C until DNA extraction. After 4 weeks of exposure, the piglets were fasted overnight before being subjected to electrical stunning and euthanized by exsanguination. The pH was measured in the colon content. Jejunum and colon contents were collected and stored at −80°C until DNA extraction. A sample of colon content (1 g) was stored in H_2_SO_4_ (25% v/v) until quantification of short chain fatty acids by gas chromatography, as described before.^46^

### 16S rRNA gene amplicon sequencing and sequence analysis

The microbiota composition was analyzed as reported previously.^47^ Briefly, DNA was extracted from 50 mg of feces or jejunum or colon content with the Quick-DNA Fecal/Soil Microbe 96 Kit (ZymoResearch) according to the manufacturer’s instructions. PCR amplicons of the 16S rRNA gene V3-V4 region were sequenced by MiSeq technology (Illumina) at the Genomic and transcriptomic platform (GeT-PlaGe, INRAE, Toulouse, France). Sequencing reads were deposited in the National Center for Biotechnology Center for Biotechnology Information Sequence (accession number: PRJNA1156376). Amplicon sequences were analyzed by the FROGS pipeline version 4.1.0,^48^ following the guidelines. The taxonomic affiliation of amplicon sequence variants (ASV) was performed with the 16S SILVA database (138.1, pintail 100). The number of reads per sample was as follow: mean = 16 349, min = 5 762, max = 27 801 in feces; mean = 21 271, min = 10 336, max = 35 984 in jejunum; mean = 15 342, min = 8 077, max = 25 476 in colon. For α and β-diversity analyses, the count table was rarefied with the R software (4.2.0) and the phyloseq package. Microbiota richness (number of observed ASV), Shannon and Inverse-Simpson α-diversity indices were calculated. β-diversity was analyzed with the distance of Bray-Curtis and visualized by PCoA (feces) or nMDS (jejunum and colon) according to the quality of data representation. The unrarefied count table was used to calculate the relative abundances of bacterial taxa at the phylum, family, and genus level.

### Statistical analyses

Statistical analyses were performed in R (version 4.2.0). *Organoid data*: All data were log normalized before analysis with linear mixed models (R packages car, lme4, emmeans).^49–51^ For repeated measurement of TEER, fixed effects included treatment, time and their interaction. Random effects included the pig from which organoids were derived and the well. Groups were compared pairwise at each time point with the Tukey post-hoc test. For all other measurements, the fixed effect was the treatment, the random effect was the pig, and groups were compared pairwise with the Tukey post-hoc test. Principal components analyses (PCA) were performed with the mixOmics package on the log normalized expression data of the 68 genes analyzed.^52^ For graphical representations, all results were expressed relatively to the control (PBS). *In vivo data*: The microbiota structure was compared between groups with a PERMANOVA using the adonis2 function of the R vegan package. Concentration of short chain fatty acids, pH, diversity indices and bacterial taxa which relative abundance was above the repeatability threshold of 0.5%^53^ were compared between groups with a non-parametric Kruskal-Wallis test. Benjamin-Hochberg procedure was used to calculate false discovery rate adjustment of p-values in order to control for multiple testing of relative abundances of bacterial families and genera.

## Results

### Deoxynivalenol dose-dependently disrupts the epithelial barrier integrity

**Figure 1.**
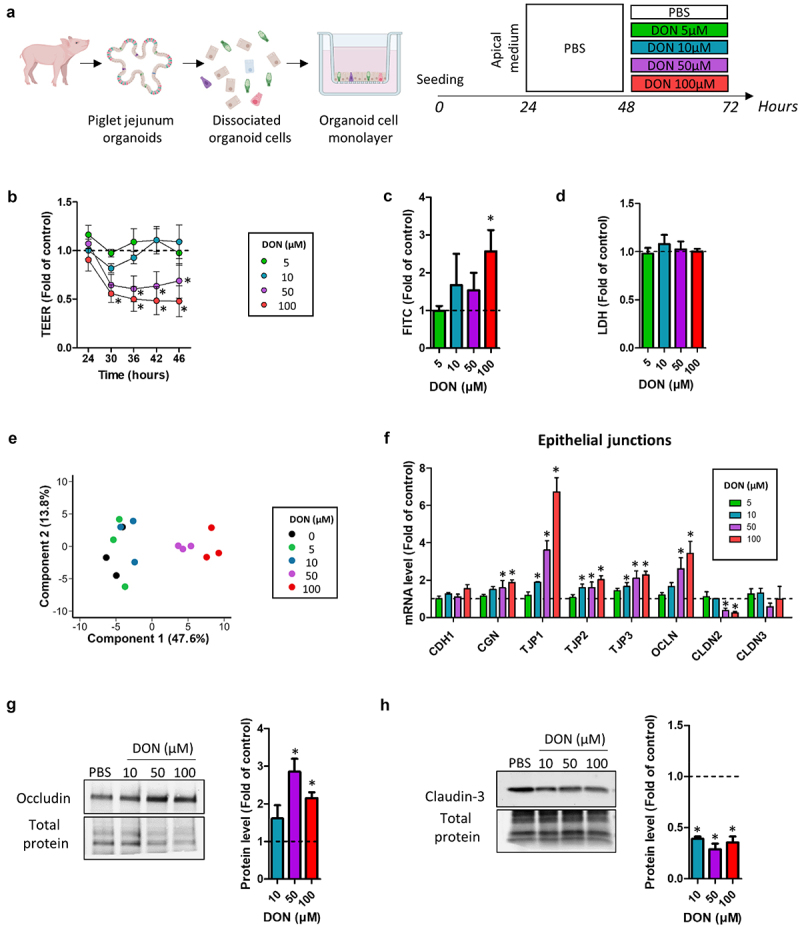
Deoxynivalenol dose-dependently disrupts the epithelial barrier integrity.

Our first objective was to evaluate concentration-dependent effects of DON in our model of cell monolayers derived from pig jejunum organoids. To this end, we exposed epithelial cells at the apical side for 24 h to 4 concentrations of DON (5, 10, 50 and 100 µM), based on previous studies in diverse *in vitro* and *ex vivo* intestinal models^6^ (Figure 1(a)). DON 50 µM and 100 µM rapidly disrupted the epithelial barrier integrity as indicated by TEER measurement (Figure 1(b)). In contrast, lower concentrations of DON (5 and 10 µM) had no effect on the epithelial barrier integrity. Measurement of transepithelial passage of FITC-dextran 4 kDa after 24 h of treatment indicated that only DON 100 µM induced a significant increase in paracellular permeability (Figure 1(c)). None of the concentrations of DON tested induced toxicity, as indicated by the measurement of LDH released in the apical medium after 24 h of treatment (Figure 1(d)).

Cell monolayers derived from jejunum organoids of suckling piglets were treated at the apical side with PBS (control) or with deoxynivalenol (DON) at 5, 10, 50 or 100 µM for 24 h. a) Schematic representation of the experimental design. b) Transepithelial electrical resistance (TEER). Data are represented as means ± SEM, *n* = 6/group. c) Paracellular permeability to FITC-dextran 4 kDa. Data are represented as means ± SEM, *n* = 6/group. d) Lactate dehydrogenase (LDH) level in the apical medium. Data are represented as means ± SEM, *n* = 3/group. e) Principal component analysis based on the expression of 68 genes. Each dot represents one sample (*n* = 3/group). Principal components 1 and 2 are shown and the corresponding percentages of explained variance are indicated. f) Relative expression of genes coding for proteins involved in the formation of cellular junctions. Data are represented as means ± SEM, *n* = 3/group. g) Western blot of occludin (OCLN) and total protein used as a loading control. Data are represented as means ± SEM, *n* = 4/group. h) Western blot of claudin-3 (CLDN3) and total protein used as a loading control. Data are represented as means ± SEM, *n* = 4/group. All data are expressed relatively to the control condition (PBS), represented by the dotted line. *: significant difference with the control (*p* < 0.05).

We analyzed the gene expression of 68 genes coding for markers of the main functions and cell populations of the intestinal epithelium. PCA revealed that DON 50 µM and 100 µM altered transcription profiles in pig jejunum organoid cells (Figure 1(e)). In contrast, the global gene expression profile of cells treated with lower concentrations of DON (5 µM and 10 µM) was similar to control cells. In order to further characterize the disruption of the epithelial barrier integrity induced by DON 50 µM and 100 µM, we first focused our analyses on genes coding for proteins involved in cellular junctions. We found that DON 10, 50 and 100 µM increased the gene expression of the tight junction proteins TJP1, TJP2 and TJP3 (Figure 1(f)). Moreover, DON 50 and 100 µM also upregulated the expression of CGN and OCLN while it reduced the expression of the pore-forming claudin-2 (CLDN2) (Figure 1(f)). Western blot analysis confirmed at the protein level that DON 50 µM and 100 µM increased the abundance of OCLN (Figure 1(g)). We also observed that DON (10–100 µM) strongly reduced the protein level of claudin-3 (Figure 1(h)), although we found no significant changes at the gene expression level (Figure 1(f)). Overall, our data show that a high concentration of DON disrupted the epithelial barrier integrity in cell monolayers derived from pig jejunum organoids.

### Deoxynivalenol dose-dependently alters epithelial renewal and differentiation

**Figure 2.**
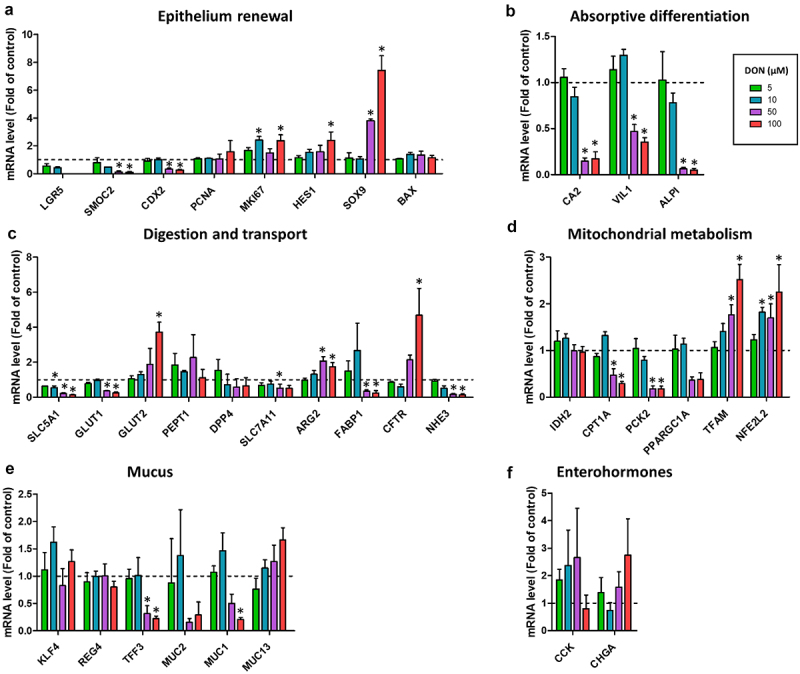
Deoxynivalenol dose-dependently alters the expression of genes involved in epithelial proliferation and differentiation.

We took advantage of the multicellular composition of pig jejunum organoids that are derived from stem cells to evaluate the concentration-dependent effects of DON on the populations of epithelial cells. The gene expression of the stem cell marker LGR5 was very low or undetected in cells treated with DON 50 µM and 100 µM (Figure 2(a)). DON 50 µM and 100 µM also reduced the gene expression of the stem cell marker SMOC2 and of CDX2. In contrast, DON 10 µM and 100 µM increased the expression of the proliferation marker MKI67. The increased gene expression of HES1 induced by DON 100 µM indicated an activation of the NOTCH signaling pathway in epithelial cells. DON 50 µM and 100 µM also strongly upregulated the expression of the progenitor cell marker SOX9. These results suggest that the highest concentrations of DON tested depleted the stem cell pool while promoting the population of proliferative progenitors.

Cell monolayers derived from jejunum organoids of suckling piglets were treated at the apical side with PBS (control) or with deoxynivalenol (DON) at 5, 10, 50 or 100 µM for 24 h. a–f) Relative gene expressions are represented as means ± SEM, *n* = 3/group. a) Stem cell and epithelial proliferation markers. The expression of LGR5 was not detected in 2/3 samples in the 50 µM DON group and in 1/3 samples in the 100 µM DON group. b) Absorptive enterocyte differentiation markers. c) Genes involved in nutrient absorption, digestion and electrolyte secretion. d) Genes involved in mitochondrial functions. e) Genes involved in mucus production. f) Enteroendocrine cell markers. All data are expressed relatively to the control condition (PBS), represented by the dotted line. *: significant difference with the control (*p* < 0.05).

DON 50 µM and 100 µM strongly reduced the differentiation of absorptive cells, as indicated by the lower gene expression of mature enterocyte markers (CA2, VIL1, ALPI) (Figure 2(b)). Accordingly, DON 50 µM and/or 100 µM reduced the expression of genes involved in the transport of nutrients and electrolytes (SLC5A1, GLUT1, SLC7A11, FABP1, NHE3) (Figure 2(c)). In contrast, DON 100 µM increased the expression of the glucose transporter GLUT2 and of the ion channel CFTR. DON 50 µM and 100 µM also upregulated the expression of Arginase 2 (ARG2), suggesting a metabolic shift in enterocytes. Indeed, DON 50 µM and 100 µM strongly reduced the expression of genes involved in mitochondrial metabolism (CPT1A, PCK2) while it increased the gene expression of master regulators of mitochondrial function (TFAM, NFE2L2) (Figure 2(d)). Regarding goblet cells, DON 50 µM and 100 µM reduced the gene expression of TFF3, coding for a peptide secreted in mucus (Figure 2(e)). DON 100 µM also reduced the expression of the glycocalyx-forming mucin MUC1. Gene expression of enteroendocrine cell makers remained unchanged after DON exposure (Figure 2(f)). Altogether, our results indicate that DON dose-dependently reduced the differentiation of enterocytes and altered the metabolism and absorptive functions of the jejunum epithelium.

### Deoxynivalenol dose-dependently disrupts epithelial antimicrobial defenses

**Figure 3.**
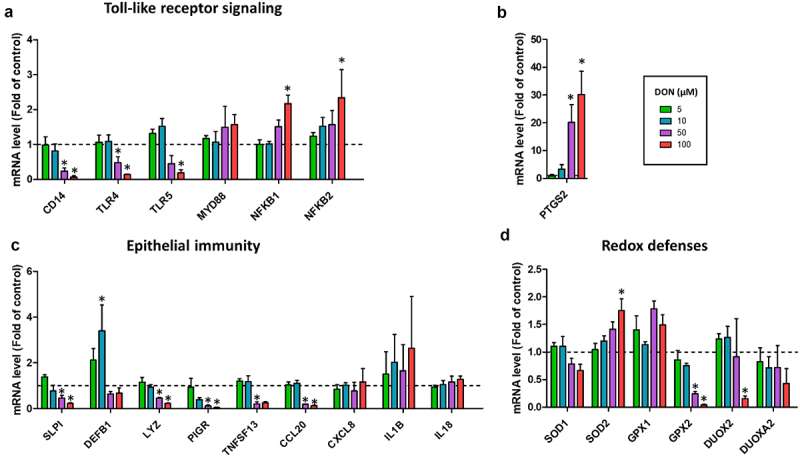
Deoxynivalenol dose-dependently alters the expression of genes involved in epithelial innate immunity.

As a next step, we examined the dose-dependent effects of DON on antimicrobial defenses, which is a major function of the intestinal epithelium. DON 50 µM and/or 100 µM reduced the expression of genes involved in Toll-like receptors (TLR) signaling (CD14, TLR4, TLR5) while increasing the expression of the NF-κB subunits (NFKB1, NFKB2) (Figure 3(a)). The gene expression of the pro-inflammatory enzyme prostaglandin synthase 2 (PTGS2, also known as COX-2) was strongly induced by DON 50 µM (20-fold increase) and 100 µM (30-fold increase) (Figure 3(b)). In contrast, DON 50 µM and 100 µM reduced the gene expression of antimicrobial peptides (SLPI, LYZ), proteins involved in immunoglobulin secretion (PIGR, TNFSF13) and a cytokine (CCL20) (Figure 3(c)). The gene expression of the antimicrobial peptide DEFB1 was upregulated only by DON 10 µM (Figure 3(c)). Additionally, DON 100 µM altered redox balance as indicated by the higher gene expression of SOD2 and the lower expression of GPX2 and DUOX2 (Figure 3(d)). GPX2 was also DON downregulated by DON 50 µM. Taken as a whole, our data show that DON disrupt the epithelial defense systems in cell monolayers derived from pig jejunum organoids.

Cell monolayers derived from jejunum organoids of suckling piglets were treated at the apical side with PBS (control) or with deoxynivalenol (DON) at 5, 10, 50 or 100 µM for 24 h. a–d) Relative gene expressions are represented as means ± SEM, *n* = 3/group. a) Genes involved in Toll-like receptor (TLR) signaling. The expression of CD14 was not detected in 1/3 samples in the 100 µM DON group. b) Prostaglandin-2 (also known as cyclooxygenase 2). c) Antimicrobial peptides, cytokines and genes involved in immunoglobulin secretion. The expression of IL1B was not detected in 1/3 samples in the 100 µM DON group. d) Genes involved in redox balance. All data are expressed relatively to the control condition (PBS), represented by the dotted line. *: significant difference with the control (*p* < 0.05).

### Butyrate attenuates the disruption of epithelial barrier integrity induced by deoxynivalenol

**Figure 4.**
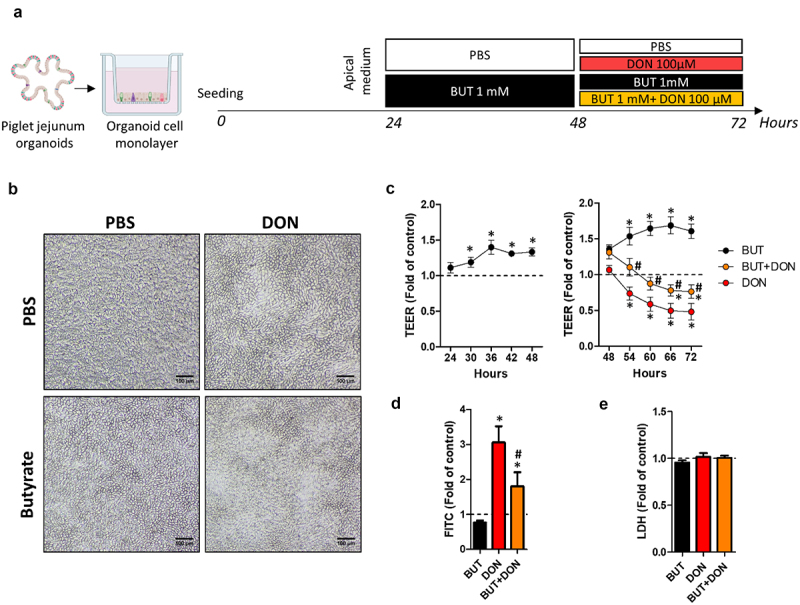
Butyrate alleviates deoxynivalenol-induced disruption of epithelial barrier integrity.

Our results indicated that, in our pig jejunum organoid cell monolayer model, DON disrupted epithelial barrier integrity, increased permeability, reduced absorptive cell differentiation and impaired metabolism and innate immunity. Conversely, the gut microbiota metabolite BUT is known to exert the opposite effects on intestinal epithelial cells.^25^ Thus, we reasoned that BUT could alleviate the toxic effects of DON. In order to evaluate the potential protective effect of this bacterial metabolite, we treated cell monolayers derived from pig jejunum organoids with BUT 1 mM for 24 h (Figure 4(a)), this concentration being used in other studies in intestinal organoids^29^ and corresponding to a realistic concentration in the jejunal content.^54^ Then, cells were exposed to DON (100 µM, this concentration was able to increase paracellular epithelial permeability to FITC-dextran 4 kDa in our model) in combination or not with BUT. The jejunum organoid cell monolayers remained confluent at the end of the treatments in all conditions, although DON induced some heterogeneity in the morphology of cells (Figure 4(b)). As expected, the 24 h pre-treatment with BUT increased the epithelial barrier sealing, according to TEER measurement (Figure 4(c)). TEER remained higher than control until 48 h when cells were exposed to BUT only. BUT attenuated the decrease in TEER induced by DON. BUT also reduced the increase in paracellular permeability triggered by DON, as revealed by analysis of the apical-to-basal transport of FITC-dextran 4 kDa (Figure 4(d)). Treatments induced no toxicity according to the level of LDH released in the apical culture medium (Figure 4(e)). Overall, our results show that BUT attenuates the disruption of epithelial barrier integrity induced by DON.

Cell monolayers derived from jejunum organoids of suckling piglets were pre-treated at the apical side with PBS (control) or butyrate (BUT, 1 mM) for 24 h. Cells were then treated at the apical side with PBS (control) or deoxynivalenol (DON, 100 µM) or BUT (1 mM) or BUT+DON. a) Schematic representation of the experimental design. b) Representative observation of cell monolayers. Scale bars represent 100 µm. c) Transepithelial electrical resistance (TEER). Data are represented as means ± SEM, *n* > 19/group. d) Paracellular permeability to FITC-dextran 4 kDa. Data are represented as means ± SEM, *n* > 10/group. e) Lactate dehydrogenase (LDH) level in the apical medium. Data are represented as means ± SEM, *n* > 11/group. All data are expressed relatively to the control condition (PBS), represented by the dotted line. *: significant difference with the control, ^#^: significant difference between the groups DON and BUT+DON (*p* < 0.05).

### Butyrate prevents the disruption of epithelial barrier integrity induced by the ribotoxin anisomycin

**Figure 5.**
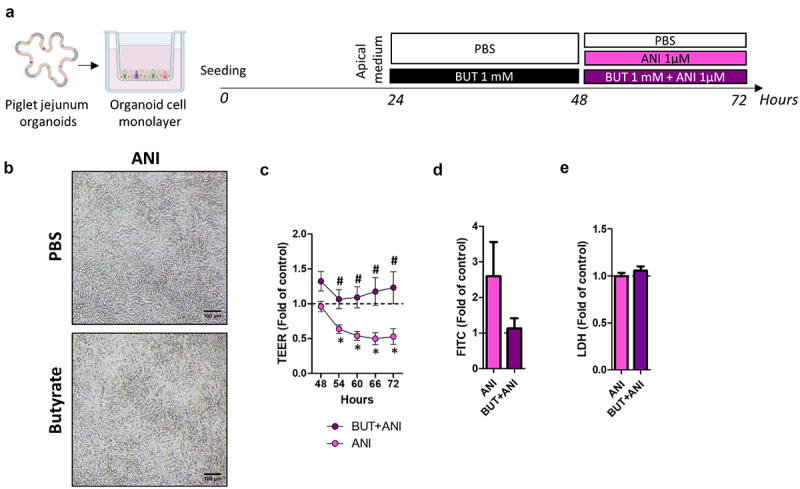
Butyrate alleviates the disruption of epithelial barrier integrity induced by the ribotoxin anisomycin.

Ribotoxicity is known to be involved in the disruption of the epithelial barrier integrity induced by DON.^4^ Thus, we searched whether BUT could alleviate the impairment of epithelial barrier integrity induced by DON through counteracting its action on ribosomes. To test this hypothesis, we treated pig jejunum organoid cell monolayers with anisomycin (ANI, 1 µM), another known ribotoxin, in combination or not with BUT (Figure 5(a)). Cell monolayers remained confluent after treatment with ANI (Figure 5(b)). Similar to DON, ANI disrupted the epithelial barrier integrity, as indicated by TEER measurement (Figure 5(c)), despite failing to induce a significant increase in paracellular permeability, as evaluated by FITC-dextran 4 kDa assay (Figure 5(d)). BUT prevented the disruption of epithelial barrier integrity induced by ANI (Figure 5(c)). Treatments were not cytotoxic according to the measurement of LDH (Figure 5(e)). We concluded that BUT is able to reduce the disruption of epithelial barrier integrity induced by ribotoxins, such as DON and ANI.

Cell monolayers derived from jejunum organoids of suckling piglets were pre-treated at the apical side with PBS (control) or butyrate (BUT, 1 mM) for 24 h. Cells were then treated at the apical side with PBS (control) or anisomycin (ANI, 1 µM) or BUT+ANI. a) Schematic representation of the experimental design. b) Representative observation of cell monolayers. Scale bars represent 100 µm. c) Transepithelial electrical resistance (TEER). Data are represented as means ± SEM, *n* = 10/group. d) Paracellular permeability to FITC-dextran 4 kDa. Data are represented as means ± SEM, *n* = 8/group. e) Lactate dehydrogenase (LDH) level in the apical medium. Data are represented as means ± SEM, *n* = 11/group. All data are expressed relatively to the control condition (PBS), represented by the dotted line. *: significant difference with the control, ^#^: significant difference between the groups ANI and BUT+ANI (*p* < 0.05).

### Butyrate alleviates the alteration of tight junctions induced by deoxynivalenol

**Figure 6.**
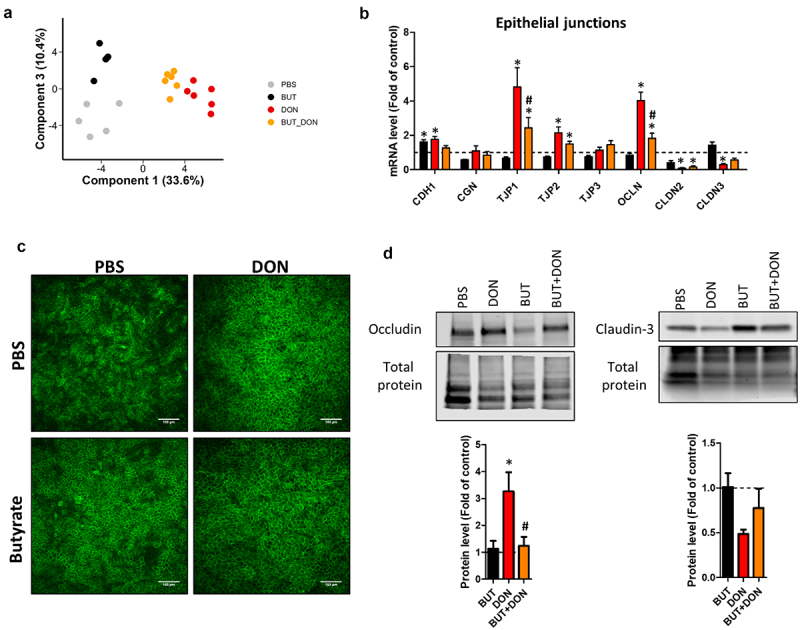
Butyrate reduces the disruption of tight junctions induced by deoxynivalenol.

In order to gain insights into the mechanisms involved in the protective effects of BUT against DON toxicity, we evaluated the expression of a panel of 68 genes in cell monolayers derived from pig jejunum organoids. PCA revealed that the effect of DON on gene expression was attenuated by BUT (PC1) and that BUT alone also modified gene expression (PC3) (Figure 6(a)). PC2 represented unidentified sources of variance (Supplementary figure S1). We first focused our analysis on cellular junctions. The upregulation of the tight junction proteins TJP1 and OCLN induced by DON was reduced by BUT (Figure 6(b)), while the effects of DON on other junction proteins were not significantly changed by BUT. Confocal microscopy imaging showed that the OCLN network was not disrupted by DON (Figure 6(c)). Western blot confirmed at the protein level that the increase abundance of OCLN induced by DON was prevented by BUT (Figure 6(d)). In contrast, no significant effects of treatments were observed for claudin-3 (Figure 6(e)). Our data indicate that the protective effects of BUT on the epithelial barrier are associated with a reduction of the effects of DON on tight junctions.

Cell monolayers derived from jejunum organoids of suckling piglets were pre-treated at the apical side with PBS (control) or butyrate (BUT, 1 mM) for 24 h. Cells were then treated at the apical side with PBS (control) or deoxynivalenol (DON, 100 µM) or BUT (1 mM) or BUT+DON. a) Principal component analysis based on the expression of 68 genes. Each dot represents one sample (*n* = 5-6/group). Principal components 1 and 3 are shown and the corresponding percentages of explained variance are indicated. Principal component 2 represent unidentified sources of variance (shown in supplemental Figure 1). b) Relative expression of genes coding for proteins involved in the formation of cellular junctions. Data are represented as means ± SEM, *n* = 5-6/group. c) Representative images of confocal microscopy observation of cell monolayers stained for occludin (OCLN). Scale bars represent 100 µm. d) Western blot of occludin (OCLN), claudin-3 (CLDN3) and total protein used as a loading control. Data are represented as means ± SEM, *n* = 4/group. All data are expressed relatively to the control condition (PBS), represented by the dotted line. *: significant difference with the control, ^#^: significant difference between the groups DON and BUT+DON (*p* < 0.05).

### Butyrate, but not deoxynivalenol, is transported across cell monolayers derived from pig jejunum organoids

**Figure 7.**
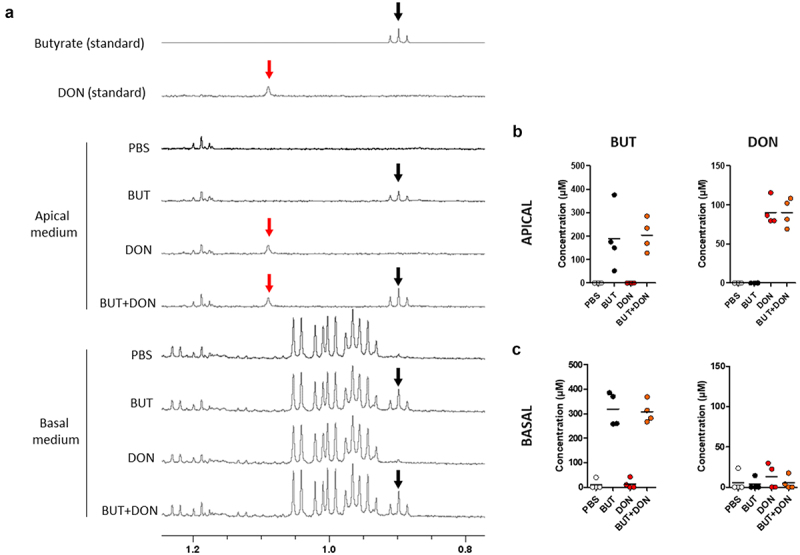
Butyrate, but not deoxynivalenol, is transported across cell monolayers derived from pig intestinal organoids.

We next asked if the improvement of the barrier integrity induced by BUT could reduce the transepithelial passage of DON toward the basal side. To this end, we analyzed by NMR spectroscopy the apical and basal media of cell monolayers collected at the end of the experiments. Our results show that BUT concentration was lower than 400 µM in the apical medium after 48 h of exposure, indicating a reduction of the initial 1 mM BUT concentration (Figures 7(a,b)). In contrast, the DON apical concentration remained close to the initial 100 µM concentration used for treatments (Figures 7(a,b)). At the basal side, BUT concentration was in the range of 200 and 400 µM, while DON was not reproducibly detected in the basal medium (Figures 7(a,c)). These results indicate that DON is not transported through the monolayer of cells derived from pig jejunum organoids in our experimental conditions.

Cell monolayers derived from jejunum organoids of suckling piglets were pre-treated at the apical side with PBS (control) or butyrate (BUT, 1 mM) for 24 h. Cells were then treated at the apical side with PBS (control) or deoxynivalenol (DON, 100 µM) or BUT (1 mM) or BUT+DON. a) Representative nuclear magnetic resonance spectra (0.8 – 1.3 ppm region) of pure BUT (black arrows, triplet @0.89ppm) or DON (red arrows, singulet @1.09ppm) and of apical and basal media collected at the end of the experiments. b) Concentrations of BUT and DON in the apical medium. Data are represented as individual values (dots) and means (bars), *n* = 4/group. c) Concentrations of BUT and DON in the basal medium. Data are represented as individual values (dots) and means (bars), *n* = 4/group.

### Butyrate attenuates the detrimental effects of deoxynivalenol on epithelial differentiation

**Figure 8.**
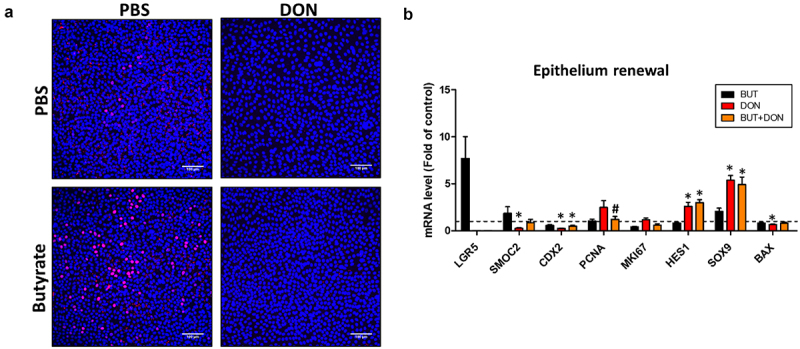
Butyrate does not prevent the effects of DON on epithelial proliferation.

Since BUT is known to regulate cell fates in the intestinal epithelium^24^, we evaluated whether this bacterial metabolite could reduce the perturbation of epithelial proliferation and differentiation induced by DON. EdU staining indicated that BUT increased epithelial proliferation (Figure 8(a)), which was associated with a non-significant increased expression of the stem cell marker LGR5 (Figure 8(b)). In contrast, no proliferation was observed when cells were treated with DON (Figure 8(a)). The expression of the proliferation marker PNCA was lower when BUT was combined with DON, when compared to DON (Figure 8(b)), while other effects of DON on the expression of genes involved in epithelial renewal were not significantly altered by BUT. These results indicate that BUT does not alleviate the effects of DON on epithelial stem and progenitor cells.

Cell monolayers derived from jejunum organoids of suckling piglets were pre-treated at the apical side with PBS (control) or butyrate (BUT, 1 mM) for 24 h. Cells were then treated at the apical side with PBS (control) or deoxynivalenol (DON, 100 µM) or BUT (1 mM) or BUT+DON. a) Representative images of confocal microscopy observation of cell monolayers stained by EdU for 2 h (red, indicating proliferative cells). DAPI staining (blue) shows nuclei. Scale bars represent 100 µm. b) Relative expression of genes involved in epithelial renewal and proliferation. The expression of LGR5 was not detected in 1/6 samples in the BUT group, 5/6 in the DON group and 3/6 in the BUT+DON group. The expression of SMOC2 was not detected in 1/6 samples in the DON group. Data are represented as means ± SEM, *n* = 5-6/group. All data are expressed relatively to the control condition (PBS), represented by the dotted line. *: significant difference with the control, ^#^: significant difference between the groups DON and BUT+DON (*p* < 0.05).

**Figure 9.**
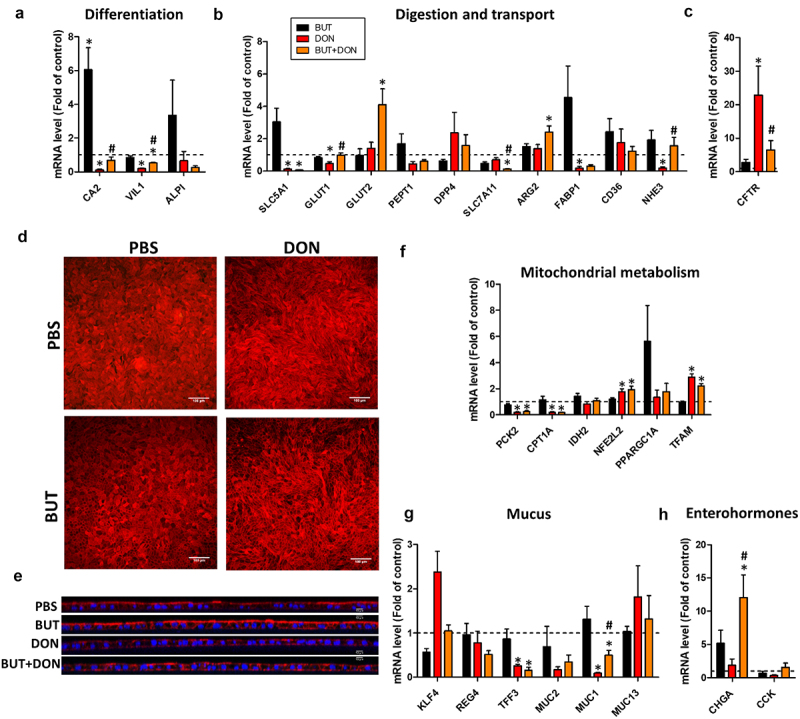
Butyrate alleviates the effects of DON on epithelial differentiation.

BUT strongly upregulated the gene expression of CA2 (6-fold increase) (Figure 9(a)), which suggests an increased epithelial differentiation toward the absorptive lineage. Accordingly, BUT alleviated the DON-induced reduction of the gene expression of markers indicating absorptive cell differentiation (CA2, VIL1) (Figure 9(a)) and of epithelial transporters (GLUT1, NHE3) (Figure 9(b)). Additionally, BUT prevented the strong upregulation of CFTR induced by DON (Figure 9(c)). Observation of actin cytoskeleton by confocal imaging revealed that DON induced cell elongation while BUT seemed to restore the geometrical shape that characterizes differentiated epithelial cells (Figure 9(d)). BUT also seemed to attenuate the DON-induced disruption of cell polarity, as seen at the apical side of epithelial cells where actin accumulation corresponds to microvilli (Figure 9(e)). However, BUT did not prevent the effects of DON on the expression of other genes involved in epithelial transport or metabolism (Figure 9(b,f)). Regarding the mucus barrier, BUT alleviated the DON-induced reduction of MUC1 expression, while it did not counteract the effect of DON on the expression of TFF3 (Figure 9(g)). BUT also strongly upregulated the gene expression of the enteroendocrine cell marker CHGA in cells treated with DON (Figure 9(h)). Altogether, these results indicate that BUT counteracted part of the detrimental effects of DON on epithelial cell differentiation.

Cell monolayers derived from jejunum organoids of suckling piglets were pre-treated at the apical side with PBS (control) or butyrate (BUT, 1 mM) for 24 h. Cells were then treated at the apical side with PBS (control) or deoxynivalenol (DON, 100 µM) or BUT (1 mM) or BUT+DON. a–c and f–h) Relative gene expressions are represented as means ± SEM, *n* = 5-6/group. a) Absorptive enterocytes differentiation markers. The expression of ALPI was not detected in 1/6 samples in the DON group and in 1/6 samples in the BUT+DON group. b) Genes involved in nutrient absorption, digestion and electrolyte secretion. The expression of FABP1 was not detected in 1/6 samples in the BUT+DON group. The expression of NHE3 was not detected in 1/6 samples in the DON group and in 1/6 samples in the BUT+DON group. c) Cystic Fibrosis Transmembrane Conductance Regulator. f) Genes involved in mitochondrial functions. g) Genes involved in mucus production. The expression of MUC2 was not detected in 1/6 samples in the DON group. h) Enteroendocrine cell markers. The expression of CCK was not detected in 1/6 samples in the DON group. d) Representative images of confocal microscopy observation of cell monolayers stained for actin. Scale bars represent 100 µm. e) Cross-section observation of cell monolayers stained for actin (red) and nuclei (blue). Scale bars represent 10 µm. All data are expressed relatively to the control condition (PBS), represented by the dotted line. *: significant difference with the control, ^#^: significant difference between the groups DON and BUT+DON (*p* < 0.05).

### Butyrate alleviates the disruption of epithelial defenses induced by deoxynivalenol

**Figure 10.**
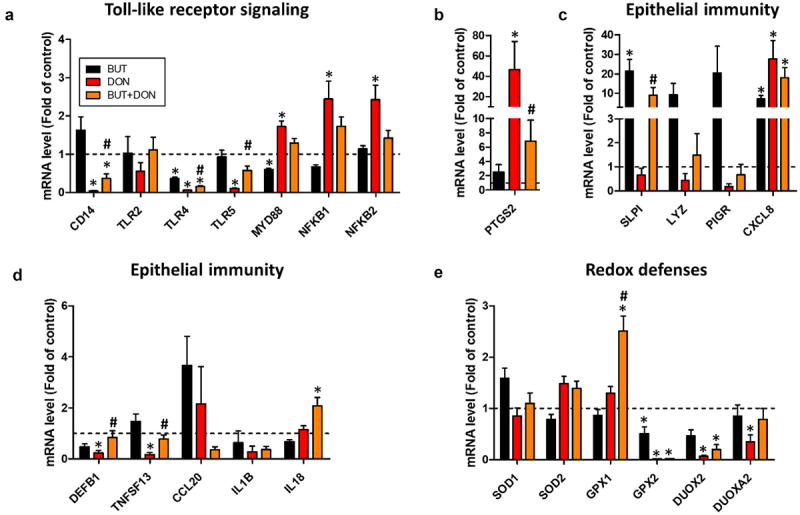
Butyrate reduces the don-induced disruption of epithelial defenses.

As a next step, we evaluated whether BUT was able to attenuate the effects of DON on epithelial innate defense systems. BUT alleviated the DON-induced downregulation of genes involved in TLR signaling (CD14, TLR4 and TLR5) (Figure 10(a)). In contrast, BUT failed to significantly counteract the upregulation of MYD88, NFKB1 and NFKB2 induced by DON. The increase in expression of the inflammatory enzyme PTGS2 induced by DON was efficiently attenuated by BUT (Figure 10(b)). In order to confirm this result, we attempted to quantify prostaglandin E2 using ELISA in the apical and basal media but its concentration was below the detection level in all conditions (data not shown). BUT strongly upregulated the gene expression of the antimicrobial peptide SLPI and this effect was also observed when cells were treated with DON (Figure 10(c)). Both BUT and DON induced the expression of the cytokine CXCL8 (Figure 10(c)). This result could not be confirmed at the protein level as CXCL8 concentration measured by ELISA in the apical and basolateral media was below the detection threshold in all conditions (data not shown). BUT prevented the DON-induced downregulation of the antimicrobial peptide DEFB1 and of the B-cell inducing chemokine TNFSF13 (Figure 10(d)). Surprisingly, BUT and DON synergistically upregulated the expression of the cytokine IL18 and of the antioxidant enzyme GPX1 (Figures 10(d,e)). The DON-induced downregulation of other genes involved in redox defenses was not prevented by BUT (Figure 10(e)). Overall, our results show that BUT partly restored the epithelial defenses disrupted by DON.

Cell monolayers derived from jejunum organoids of suckling piglets were pre-treated at the apical side with PBS (control) or butyrate (BUT, 1 mM) for 24 h. Cells were then treated at the apical side with PBS (control) or deoxynivalenol (DON, 100 µM) or BUT (1 mM) or BUT+DON. a–e) Relative gene expressions are represented as means ± SEM, *n* = 5-6/group. a) Genes involved in Toll-like receptor (TLR) signaling. The expression of CD14 was not detected in 1/6 samples in the DON group. The expression of TLR2 was not detected in 1/6 samples in the BUT group, 1/6 samples in the DON group and 2/6 samples in the BUT+DON group. The expression of TLR5 was not detected in 1/6 samples in the DON group. b) Prostaglandin-2 (also known as cyclooxygenase 2). c,d) Antimicrobial peptides, cytokines and genes involved in immunoglobulin secretion. The expression of LYZ was not detected in 1/6 samples in the BUT group. The expression of PIGR was not detected in 2/6 samples in the DON group. The expression of TNFSF13 was not detected in 1/6 samples in the DON group. The expression of IL1B was not detected in 1/6 samples in the BUT group and 1/6 samples in the DON group. e) Genes involved in redox defenses. The expression of DUOX2 was not detected in 1/6 samples in the DON group and 2/6 samples in the BUT+DON group. All data are expressed relatively to the control condition (PBS), represented by the dotted line. *: significant difference with the control, ^#^: significant difference between the groups DON and BUT+DON (*p* < 0.05).

### Inhibition of histone deacetylases reproduces some of the effects of butyrate on gene expression

**Figure 11.**
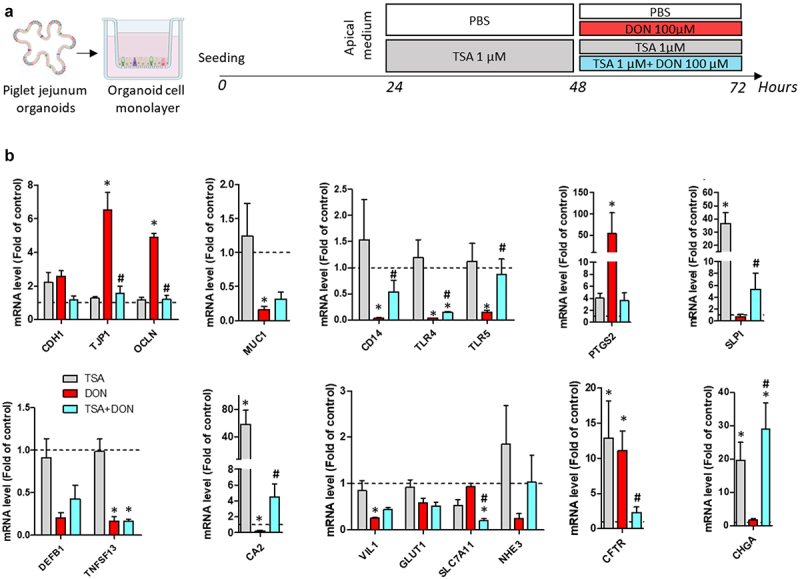
Inhibition of histone deacetylases reproduces some of the effects of butyrate on gene expression.

Inhibition of histone deacetylases (HDAC) is one of the mode of action through which butyrate influences gene expression in intestinal epithelial cells.^24^ To evaluate the relevance of this potential mode of action in our model, we treated pig jejunum organoid cell monolayers with trichostatin A (TSA, 1 µM), another HDAC inhibitor, in combination or not with DON (Figure 11(a)). Cell monolayers remained confluent after TSA and DON treatment, while the combination of TSA+DON detached the cells from the culture insert in 3 out of 6 replicates. Although we did not identify the reason underlying the experimental variability in this condition, we performed gene expression analysis on the 3 replicates that remained confluent. We focused our analysis on selected genes that we previously found regulated by BUT in order to evaluate whether TSA reproduced its effects (Figure 11(b)). TSA alone strongly increased the gene expression of SLPI and CA2, as observed before with BUT. TSA also reproduced the BUT-induced attenuation of the effects of DON on the gene expression of TJP1, OCLN, CD14, TLR4, TLR5, CA2, and CFTR. Contrary to BUT, TSA failed to significantly prevent the effects of DON on the gene expression of MUC1, PTGS2, TNFSF13, VIL1. Finally, the combination of TSA+DON reproduced the increased expression of SLPI and CHGA previously observed with BUT+DON. These results indicate that HDAC inhibition replicates some of the effects of BUT on gene expression in pig jejunum organoid cell monolayers and may partly contribute to the protective effects of BUT against DON.

Cell monolayers derived from jejunum organoids of suckling piglets were pre-treated at the apical side with PBS (control) or trichostatin A histone deacetylase inhibitor (TSA, 1 µM) for 24 h. Cells were then treated at the apical side with PBS (control) or deoxynivalenol (DON, 100 µM) or TSA (1 µM) or TSA+DON. a) Schematic representation of the experimental design. b) Relative gene expressions are represented as means ± SEM, *n* = 3/group. Genes were selected based on the previous experiments showing an effect of butyrate on their expression. All data are expressed relatively to the control condition (PBS), represented by the dotted line. *: significant difference with the control, ^#^: significant difference between the groups DON and TSA+DON (*p* < 0.05).

### BUT production by the gut microbiota is not altered by DON

**Figure 12.**
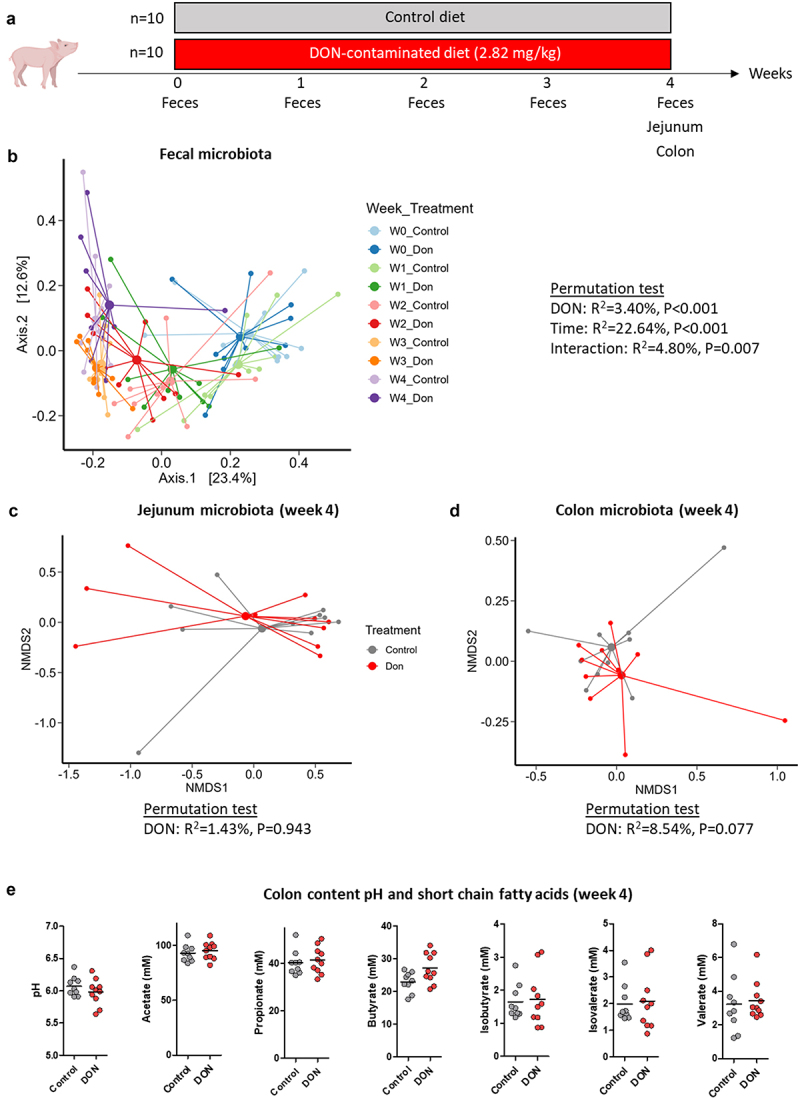
The effect of DON on the gut microbiota is moderate and transient.

As we found that BUT was able to prevent some of the toxic effects of DON *in vitro*, we next asked whether DON could disrupt the microbiota composition *in vivo* and its production of short chain fatty acids, including BUT. Thus, we evaluated in pigs the consequences of a 1-month ingestion of a DON-contaminated diet (2.82 mg/kg) on the gut microbiota in feces for longitudinal sampling and in jejunum and colon contents at slaughter (Figure 12(a)). The main factor influencing the fecal microbiota β-diversity was the age of piglet (22.6% of explained variance, Permutation test: *p* < 0.001) while we found lower effect of DON (3.4% of explained variance, *p* < 0.001) and of the interaction of DON exposure with time (4.8% of explained variance, *p* = 0.007) (Figure 12(b)). As expected, the fecal microbiota β-diversity was not different between groups at baseline (week 0, *p* = 0.330). After 1 week of exposure, DON altered the fecal microbiota β-diversity (15% of explained variance, *p* = 0.004) and reduced the microbiota richness and the relative abundance of Prevotella_7 (Supplementary table S2). After 2 weeks, DON altered the fecal microbiota β-diversity (10.2% of explained variance, *p* = 0.009) and increased the relative abundances of Clostridium sensu stricto 1 and Terrisporobacter while it reduced the relative abundance of Prevotellaceae NK3B31 group. After 3 weeks, DON altered the fecal microbiota β-diversity (13.5% of explained variance, *p* = 0.001) and increased the relative abundance of Clostridium sensu stricto 1 while it decreased the relative abundances of Streptococcus and [Eubacterium] ruminantium group. After 4 weeks, DON had no effect on the fecal microbiota β-diversity (*p* = 0.105) while it reduced the relative abundance of Streptococcus. These results indicate that DON induced moderate and transient modifications of the fecal microbiota. In agreement with the results obtained in fecal samples after 4 weeks of exposure, we found no significant effect of DON on the jejunum and colon microbiota structure, diversity and taxonomic composition (Figure 12(c,d), Supplementary table S2). Moreover, we found that content pH and the concentration of short chain fatty acids, including BUT, remained unchanged in the colon content after 4 weeks of DON exposure (Figure 12(e)). Overall, our results indicate that the ingestion of a DON-contaminated diet had no lasting effects on the gut microbiota and on its production of BUT in pigs.

Piglets were fed for 28-days either with a control diet (*n* = 10) or with a deoxynivalenol-contaminated diet (2.82 mg DON/kg of feed, *n* = 10). a) Schematic representation of the experimental design. b) Principal coordinates analysis (PCoA) two-dimensional representation of the fecal microbiota using the Bray-Curtis distance at baseline (week 0, W0) and after 1, 2, 3 or 4 weeks of exposure. c) Non-metric dimensional scaling (nMDS) two-dimensional representation of the jejunum microbiota at week 4 using the Bray-Curtis distance. Stress = 0.07. d) nMDS two-dimensional representation of the colon microbiota at week 4 using the Bray-Curtis distance. Stress = 0.08. E) Colon content pH and short chain fatty acids concentration measured at week 4.

## Discussion

Impairment of the intestinal epithelial barrier integrity is a major toxic effect of the mycotoxin DON.^6^ Accordingly, we found that DON induced a dose-dependent disruption of the epithelial barrier integrity in cell monolayers derived from pig jejunum organoids. This effect was associated with an increased expression of several tight junction proteins, such as TJP1 and OCLN, which was also observed in other *in vitro* and *in vivo* studies.^4,22^ In contrast, we observed that DON reduced the protein level of claudin-3, as described in other studies showing that the DON-induced disruption of epithelial barrier integrity was associated with a reduced expression of tight junction proteins.^35,55^ These discrepancies could reflect the complex kinetics of epithelial response to DON or compensatory transcriptional mechanisms triggered by the reduction of protein synthesis.^5^ Our results showing that BUT attenuated the DON-induced disruption of epithelial barrier integrity and associated alterations of tight junction proteins is consistent with the known capacity of this bacterial metabolite to reduce epithelial permeability.^26–29^ The preventive effect of the microbial metabolite BUT against DON-induced gut permeability indicates an overall reduction of its intestinal toxicity.

Besides its role of physical barrier, the intestinal epithelium is also a central component of the innate immune defenses in the gut, which is known to be disrupted by DON.^56^ Sensing of microbial signals by TLR plays a major role in the defense system of the intestinal epithelium.^57^ Thus, the reduced expression of key components of this pathway induced by DON in pig jejunum organoids (CD14, TRL4 and TRL5) could impair innate defenses in the epithelium, as shown previously *in vitro* in macrophages^58^ or in mesenteric lymph nodes after oral ingestion of DON in mice.^59^ The ability of BUT to counteract this inhibition of TLR-signaling might restore the capacity of epithelial cells to respond to microbial stimuli. The strong upregulation of the pro-inflammatory enzyme PTGS2 (COX-2) induced by DON in our pig jejunum organoid model is in agreement with previous studies showing that DON increased the expression of this gene through activation of the NF-κB pathway,^14,60^ which subunits NFKB1 and NFKB2 were also upregulated by DON in our model. Activation of the NF-κB pathway may be triggered by DON-induced oxidative stress, as suggested by increased levels of the NEF2L2 mRNA which encodes for NRF2, a key transcription factor of the antioxidant defense system.^61^ The protective effects of BUT against PTGS2 upregulation is in agreement with a previous study in the HT-29 epithelial cell line.^62^ Paradoxically, BUT is also known to activate innate defense systems in the intestinal epithelium.^63^ This is confirmed in our study showing that BUT induced an upregulation of the pro-inflammatory cytokine CXCL8, which expression was also increased by DON. Additionally, the combination of BUT and DON has some synergistic effects as observed for the upregulation of the cytokine IL18 and the antioxidant enzyme GPX1. This observation is consistent with previous reports showing that BUT can amplify some epithelial responses triggered by pro-inflammatory stimuli.^64^

The use of cells derived from pig jejunum organoids allowed us to study the combined effects of BUT and DON on a complex epithelium composed of diverse cell types.^36^ This cellular diversity allowed us, for example, to characterize the effects of BUT and DON on genes expressed specifically by goblet cells (e.g. MUC2, TFF3) or enteroendocrine cells (e.g. CHGA, CCK), which would not be possible when using intestinal cell lines such as IPEC-J2 that do not express these genes.^65^ Our results indicated that DON reduced the expression of genes involved in absorptive cell differentiation and altered the gene expression of nutrient and electrolyte transporters. This is consistent with the effects of DON described in enterocyte-like cell lines, showing a reduction in differentiation and an impairment of nutrient absorption through inhibition of protein synthesis.^7,8,10^ In contrast, BUT is well known to enhance absorptive cell differentiation.^24,66,67^ Accordingly, we found that BUT partially prevented the DON-induced impairment of absorptive cell differentiation notably by restoring the gene expression level of enterocyte makers (CA2, VIL1), of the glucose transporter GLUT1, of electrolyte channels (CFTR, NHE3) and of the glycocalyx forming transmembrane mucin 1 (MUC1). In contrast, BUT failed to prevent the DON-induced reduction of the TFF3 gene expression, which is a major protein secreted by goblet cells. This DON-induced goblet cell toxicity was already observed in other studies.^68^ BUT was also inefficient to prevent the effects of DON on stem and proliferating cells, notably regarding the reduced expression of stem cell makers (LGR5, SMOC2) and activation of NOTCH signaling indicated by the upregulation of HES1, consistent with recent studies.^13,69^ This differential preventive effect of BUT according to the type of epithelial cells could be explained by the “butyrate paradox” that links the metabolic capacity of each cell type to oxidize or not BUT with the divergent biological effect of this bacterial metabolite.^24^ Alternatively, the active protein synthesis in specific cell types such as stem/proliferating cells or goblet cells producing mucus could make them particularly sensitive to DON ribotoxicity,^9,17^ which could explain why BUT failed to exert preventive effects in these types of cells when compared to differentiated absorptive cells.

Numerous modes of action have been proposed for the effects of the gut microbiota metabolite BUT on the intestinal epithelium.^25^ Our results showing that BUT is able to reduce the disruption of epithelial barrier integrity induced by the two ribotoxins DON and ANI suggest that this bacterial metabolite counteracts epithelial barrier defects triggered by the inhibition of protein synthesis.^3^ Previous studies have shown that ribotoxins with similar (ANI) or different (cycloheximide) modes of action from DON have similar biological effects in intestinal epithelial cells such as the exacerbation of DNA damages^70^ or impairment of nutrient uptake.^10^ Utilization of BUT as an energy source by epithelial cells may also be involved in the protective effects of BUT against DON.^24^ Indeed, a recent study found that BUT attenuated the barrier disruption induced by a low concentration of DON (1 µM) by promoting mitochondrial metabolism in IPEC-J2 porcine epithelial cells.^35^ However, in our organoid model, BUT did not counteract the detrimental effects of DON on the expression of genes involved in mitochondrial function. Alternatively, inhibition of histone deacetylases (HDAC) or activation of G protein-coupled receptors (GPR41, GPR43 or GPR109A) by BUT may also be involved in reducing DON toxicity.^25^ Our results showing that TSA was able to reproduce some of the BUT-induced modifications of gene expression in pig jejunum organoid cell monolayers suggest that the protective effects of BUT against DON toxicity involved inhibition of HDAC. Indeed, BUT was previously shown to alleviate DON-induced hepatic damages through modification of histone acetylation.^71,71^

Our model of cell monolayer derived from pig jejunum organoids appears relatively resistant to DON-induced toxicity since epithelial permeability was increased in the 50–100 µM range while 10-fold lower concentrations can disrupt the epithelial barrier function in intestinal explants or in cell lines.^6^ A study in Caco-2 cells showed that proliferative cells are more sensitive to DON than differentiated cells.^9^ Thus, we hypothesize that a higher differentiation level of epithelial cells in organoids when compared to cell lines could explain their relative resistance to DON-induced barrier dysfunction. The multicellular composition of intestinal organoids could also contribute to protect cells against DON, notably through the secretion of mucus by goblet cells. Additionally, our model of organoids is purely epithelial and the lack of immune cells could explain the higher sensitivity of intestinal explants that contain immune cells which are prone to trigger a pro-inflammatory response upon DON exposure.^56^ Our NMR data showed that DON did not translocate through the monolayer of cells derived from pig jejunum organoids despite increasing the paracellular permeability to FITC-dextran 4 kDa. This result is surprising since DON is known to be rapidly absorbed in pigs *in vivo*^72,73^ and in Caco-2 cells.^74,75^ However, our jejunum model does not reflect absorption processes in the stomach and proximal small intestine which play an important role in DON absorption.^73^ Moreover, DON is a substrate of the efflux transporters such as P-glycoprotein (ABCB1) and multidrug resistance associated protein 2 (ABCC2) that are expressed at the apical membrane of intestinal epithelial cells.^75,76^ Thus, we hypothesize that efflux transporters expressed by epithelial cells in monolayers derived from pig jejunum organoids could export DON at the apical side, thereby reducing its intracellular concentration and preventing its secretion in the basolateral compartment. Interestingly, BUT is able to upregulate ABCB1 in T84 intestinal epithelial cells,^77^ which could contribute to reduce the concentration of intracellular DON through enhanced secretion. Alternatively, pig jejunum organoid cells may metabolize DON and thereby limit its translocation. Additional experiments would be required to test these hypotheses. The absence of DON at the basolateral side of epithelial cells in our model could also explain the relatively high concentration needed to induce barrier dysfunction since previous studies showed that DON basolateral exposure was more toxic than apical exposure.^5,17,78,79^

Our results showing that the bacterial metabolite BUT alleviates the DON-induced toxicity suggests that providing BUT as a dietary supplement could be a promising strategy to mitigate the detrimental effects of this foodborne toxin that represents a major threat for both human and animal health. The use of BUT as a postbiotic as already proven beneficial effects for gut health.^80,81^ Importantly, orally administered BUT would likely reach the small intestine, which is the main digestive segment exposed to DON. Direct supplementation of BUT appears more promising than the use of prebiotics aiming at promoting BUT endogenous production by the small intestine microbiota because its capacity to produce BUT is much lower than observed in the colon.^82^ Probiotics could also be promising as the improvement of the gut barrier function induced by *Lactobacillus rhamnosus* GG in immunocompromised mice exposed to DON was associated with an increased production of BUT by the gut microbiota.^83^ Conversely, it would be interesting to test whether the intestinal toxicity of DON could be exacerbated when the production of BUT by the gut microbiota is reduced or when its utilization by epithelial cells is impaired, as observed in intestinal diseases.^84^ Several studies suggested that DON itself could reduce the production of BUT by the gut microbiota,^83,85^ while other reports found no effects or an increased production of BUT after DON exposure.^86,87^ In our *in vivo* experiment in pigs, the moderate and transient effects of DON on the microbiota suggested a rapid adaptation of the microbial community to the presence of the mycotoxin, which resulted in similar BUT concentration in the colon after 4 weeks of DON exposure. Additional *in vitro* experiments using anaerobic culture of gut microbiota would be helpful to investigate more precisely how DON influences the bacterial production of BUT.

In conclusion, our study has demonstrated *in vitro* that BUT alleviates DON-induced disruption of epithelial barrier integrity, differentiation, and innate immune defenses in a cell monolayer derived from pig jejunum organoids. Our results suggest that the protective effect of BUT could involve a reduction of DON-induced ribotoxic stress and the regulation of gene expression through HDAC inhibition. Therefore, our findings provide new insights into the protective role of BUT against DON-induced intestinal damages. Besides BUT, numerous metabolites produced by the gut microbiota are known to regulate the epithelial barrier function^23^ and investigation of their potential protective effects against foodborne toxins could pave the way for the development of novel preventive strategies.

## Supplementary Material

Supplemental Material

## Data Availability

All data are contained in the article and the supporting information.
